# Extracellular vesicles from mesenchymal stromal cells as a promising therapy for ARDS: a systematic review of preclinical studies

**DOI:** 10.3389/fmed.2025.1665948

**Published:** 2025-10-29

**Authors:** Samuel C. F. Couto, Miquéias Lopes-Pacheco, Vanderson Rocha, Claudia C. Dos Santos, Patricia R. M. Rocco

**Affiliations:** ^1^Laboratory of Medical Investigation in Pathogenesis and Directed Therapy in Onco-Immuno-Hematology (LIM-31), Department of Hematology and Cell Therapy, Hospital das Clínicas da Faculdade de Medicina da Universidade de São Paulo, São Paulo, Brazil; ^2^Cellular Therapy Laboratory, Fundação Pró-Sangue–Hemocentro de São Paulo, São Paulo, Brazil; ^3^Department of Pediatrics, Center for Cystic Fibrosis and Airway Disease Research (CF-AIR), Emory University School of Medicine, Atlanta, GA, United States; ^4^The Keenan Research Centre for Biomedical Science of St. Michael's Hospital, Institute of Medical Sciences and Interdepartmental Division of Critical Care, Faculty of Medicine, University of Toronto, Toronto, ON, Canada; ^5^Laboratory of Pulmonary Investigation, Carlos Chagas Filho Institute of Biophysics, Federal University of Rio de Janeiro, Rio de Janeiro, Brazil

**Keywords:** mesenchymal stromal cells, extracellular vesicles, acute respiratory distress syndrome, animal models, systematic review, inflammation

## Abstract

**Introduction:**

Mesenchymal stromal cell-derived extracellular vesicles (MSC-EVs) have emerged as a promising cell-free therapeutic strategy for acute respiratory distress syndrome (ARDS), a condition with limited effective treatment options.

**Methods:**

This systematic review synthesizes findings from 51 in vivo preclinical studies investigating the efficacy, delivery methods, mechanisms of action, and optimization strategies of MSC-EV interventions in experimental ARDS.

**Results:**

Across diverse models and etiologies, MSC-EVs consistently attenuated inflammation, improved gas exchange, and enhanced survival. Mechanistically, these benefits were largely attributed to microRNA-mediated immunomodulation, including promotion of anti-inflammatory macrophage phenotypes and improved bacterial clearance. Factors influencing therapeutic efficacy included the MSC source, EV preconditioning, timing of administration, and route of delivery.

**Discussion:**

Despite these encouraging findings, critical methodological heterogeneity limits reproducibility and translational potential. This heterogeneity is particularly evident in dose metrics (e.g., particle number versus protein content), EV quantification methods (e.g., flow cytometry versus nanoparticle tracking analysis), and timing of outcome assessment. This review underscores the growing body of preclinical evidence supporting MSC-EVs in ARDS and identifies key knowledge gaps such as optimal dosing, safety profiling, and scalable manufacturing that must be addressed to enable clinical translation.

## 1 Introduction

Acute respiratory distress syndrome (ARDS) is a life-threatening condition characterized by diffuse alveolar damage, dysregulated inflammation, and impaired gas exchange (1). Despite advances in critical care, ARDS remains a major cause of morbidity and mortality, with rates exceeding 40% in moderate-to-severe cases (2). Current management, including lung-protective ventilation, prone positioning, and conservative fluid strategies, is largely supportive, as no pharmacologic treatments have consistently demonstrated clinical efficacy (3).

Mesenchymal stromal cells (MSCs) are potential therapeutics for ARDS due to their immunomodulatory, antimicrobial, and regenerative properties (4, 5). Early-phase clinical trials (Phase I/II) have confirmed that MSC administration is safe in ARDS patients. However, therapeutic benefits have been inconsistent, and evidence of efficacy remains limited (6). Mechanistic studies indicate MSCs act primarily via paracrine signaling rather than durable engraftment or differentiation (7–10). Clinical translation is further complicated by variability in MSC sources, dosing, and delivery, as well as potential safety concerns, including microvascular occlusion and tumorigenicity.

Attention has therefore shifted toward the MSC secretome, particularly extracellular vesicles (EVs). These nanoscale, membrane-bound particles carry proteins, lipids, mRNAs, and regulatory microRNAs capable of modulating recipient cell function (11–14). MSC-derived EVs (MSC-EVs) recapitulate many therapeutic effects of MSCs, including immunomodulation, inflammation attenuation, and tissue repair promotion (15, 16). Key cargo components such as anti-inflammatory microRNAs, growth factors, and mitochondria have been implicated in these effects, highlighting complex mechanisms of action (17, 18). Importantly, MSC-EVs offer translational advantages over cellular therapies: they are non-tumorigenic, exhibit low immunogenicity, lack risk of microvascular occlusion, and can be manufactured, stored, and administered as standardized, off-the-shelf products (13, 15, 17). Advances in bioengineering and targeted delivery strategies further enhance their potential as precision therapies for lung injury (19).

Given the rapidly growing body of preclinical research on MSC-EVs in ARDS, a systematic synthesis of animal studies is essential to evaluate therapeutic efficacy, clarify underlying mechanisms, and identify factors that influence outcomes. This review provides a critical appraisal of the preclinical evidence for MSC-EV therapy in ARDS, highlighting both its translational potential and the key knowledge gaps that must be addressed to advance these interventions toward clinical application.

## 2 Materials and methods

### 2.1 Search strategy

This systematic review was conducted following the Preferred Reporting Items for Systematic Reviews and Meta-Analyses (PRISMA) 2020 guidelines to ensure methodological rigor and transparency (18). The review protocol was not registered in a public database.

The search strategy combined Medical Subject Headings (MeSH) and free-text terms related to mesenchymal stromal cells, extracellular vesicles, and acute lung injury/ARDS (e.g., “extracellular vesicles” OR “microvesicles” OR “exosomes” AND “mesenchymal stromal cells” AND “ARDS” OR “acute lung injury” OR “ALI”). Boolean operators and wildcards were adapted to each database's syntax, and no filters for species or language were applied to maximize sensitivity. Full search strings for all databases are provided in Supplementary Table S1.

The final search was conducted on April 1, 2025, and updated before manuscript submission. Retrieved citations were imported into Rayyan (Rayyan.ai, version 1.0) for automated and manual duplicate removal.

### 2.2 Eligibility criteria

**Inclusion criteria:** Original *in vivo* preclinical studies were included if they: (1) employed animal models of acute lung injury (ALI) or ARDS induced by any mechanism (e.g., LPS, acid aspiration, bacterial pneumonia, ventilator-induced lung injury); (2) used MSC-derived extracellular vesicles (EVs) as the primary therapeutic intervention; (3) reported at least one *in vivo* outcome relevant to therapeutic efficacy (e.g., survival, oxygenation, histopathology, inflammatory markers, or mechanistic endpoints); (4) were published in peer-reviewed journals between 2015 and 2025; and (5) were written in English.

**Exclusion criteria:** Studies were excluded if they: (1) were limited to *in vitro* experiments; (2) employed whole MSCs, conditioned media without EV isolation, or other non-EV cell-free products; (3) were non-original publications (e.g., reviews, meta-analyses, protocols, editorials, abstracts); (4) focused on chronic lung disease or fibrosis models; or (5) represented duplicate or overlapping data, in which case the most complete or recent dataset was retained.

### 2.3 Study selection and data extraction

Two independent reviewers (S.C. and M.L.-P.) screened titles and abstracts using Rayyan, followed by duplicate full-text screening. Discrepancies were resolved through discussion and, when necessary, by a third reviewer (P.R.). Inter-rater agreement was quantified using Cohen's kappa statistic for both screening phases. Data extraction was independently performed by the same reviewers using a pre-piloted standardized form, which was tested on a random sample of five studies to ensure clarity and consistency.

### 2.4 Data extraction

The following variables were systematically extracted from each study: (1) study characteristics, including author(s), year, country, animal species/strain, sex, and group sizes; (2) ALI/ARDS model, encompassing injury type, severity, timing, and induction method; (3) intervention details, including MSC tissue source (e.g., bone marrow, adipose, umbilical cord), EV isolation method, dose, route and timing of administration, and any preconditioning or genetic modification; (4) EV characterization, including methods such as nanoparticle tracking analysis, western blotting, and electron microscopy, following MISEV2018 guidelines; and (5) outcomes, including mortality, oxygenation, lung compliance, histology, inflammatory markers (e.g., cytokines, leukocyte infiltration), bacterial clearance, and mechanistic findings (e.g., miRNA profiles, immune modulation). Data discrepancies were resolved by discussion, and when essential information was missing, study authors were contacted when feasible.

### 2.5 Risk of bias assessment

Risk of bias in the included preclinical studies was independently assessed by two reviewers using the SYRCLE Risk of Bias (RoB) tool, an adaptation of the Cochrane Collaboration framework for animal studies. The tool evaluates key domains, including selection, performance, detection, attrition, and reporting biases, as well as other potential sources of bias. Each domain was classified as “low,” “high,” or “unclear” risk of bias. Inter-rater agreement was high, with 95% concordance and a Cohen's kappa of 0.88, and any discrepancies were resolved through discussion with a third reviewer.

### 2.6 Data synthesis and analysis

Given the substantial heterogeneity across animal species, injury models, MSC sources, EV isolation methods, dosing regimens, and outcome measures, a quantitative meta-analysis was not feasible. Instead, we conducted a structured narrative synthesis, categorizing studies by injury model (e.g., endotoxin-induced, bacterial, acid aspiration, ventilator-induced) and emphasizing model-specific outcomes and mechanistic insights. Subgroup analyses were performed according to: (1) MSC tissue source; (2) EV preconditioning or genetic modification; (3) route and timing of administration; and (4) animal species.

Although sex was extracted as a variable, its inclusion in analyses was limited due to underreporting in the majority of studies, as discussed in the Results and Discussion sections. No predefined primary outcome was established because of the exploratory nature of this review; nevertheless, mortality, oxygenation, and histological injury scores were considered key indicators of therapeutic efficacy and were prioritized in the synthesis.

### 2.7 Assessment of publication bias

Given the narrative nature of the synthesis and limited reporting of standardized effect sizes, formal quantitative assessment of publication bias (e.g., funnel plots) was not possible. Nonetheless, we qualitatively considered the potential for selective publication in our interpretation of findings.

## 3 Results

### 3.1 Search results and characteristics

**Figure 1:**
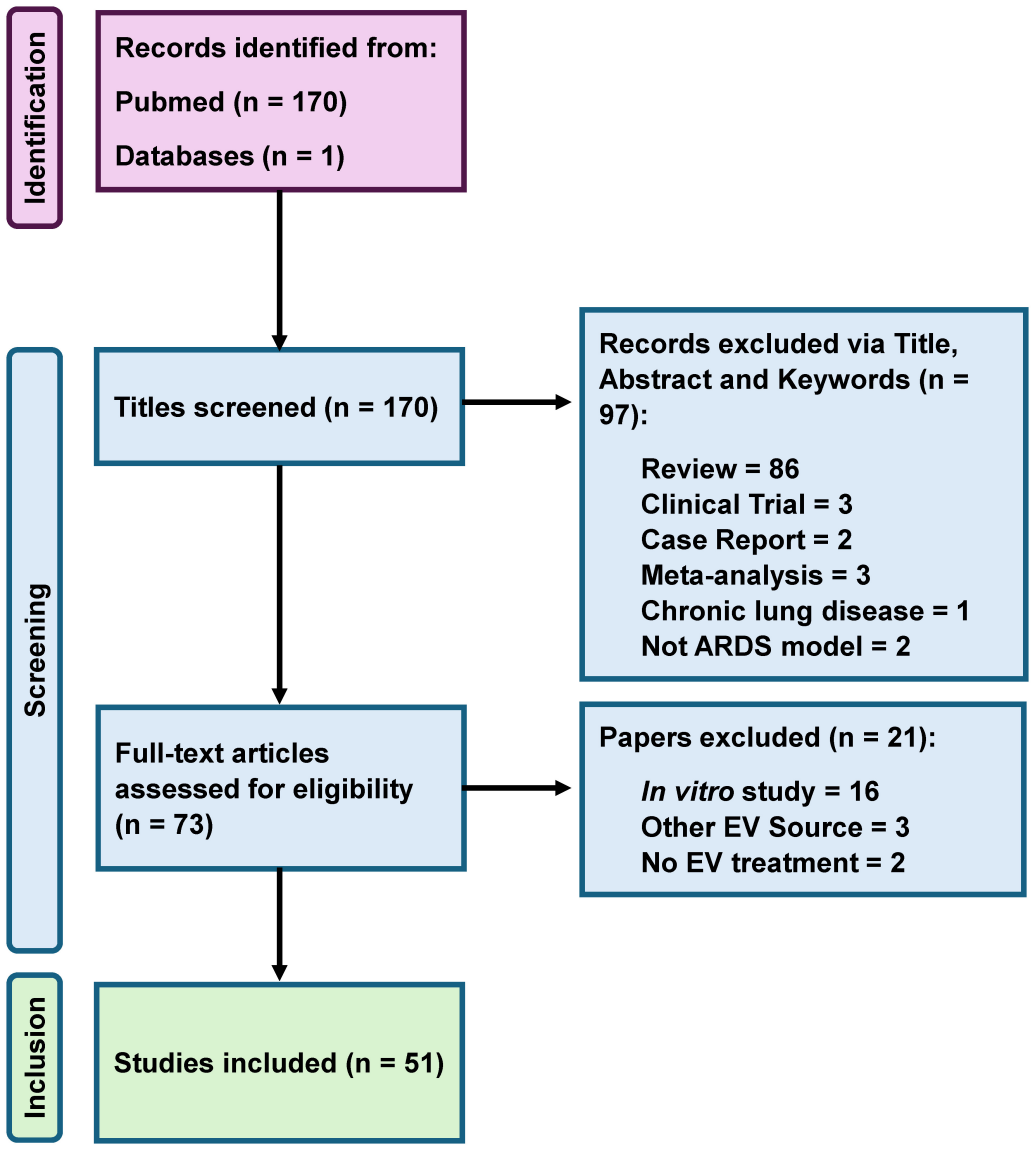
Prisma flow diagram for systematic review.

The initial database search identified 170 potentially relevant articles. After removing duplicates and screening titles and abstracts, 73 full-text articles were assessed for eligibility. Of these, 22 were excluded for not meeting predefined criteria (e.g., use of whole cells, *in vitro*-only design, or unrelated models), resulting in 51 preclinical studies being included in the final analysis (PRISMA flow diagram, Figure 1).

### 3.2 Risk of bias assessment

Overall, the risk-of-bias assessment revealed that most studies reported randomization procedures incompletely, and blinding of outcome assessment was rarely described. Allocation concealment and selective reporting were also frequently rated as “unclear” due to insufficient methodological detail. In contrast, domains related to incomplete outcome data and baseline characteristics were generally at low risk of bias. The table presents the proportion of studies classified as low, unclear, or high risk across nine methodological domains: sequence generation, baseline characteristics, allocation concealment, performance bias (random housing and caregiver/investigator blinding), detection bias (outcome assessor blinding), attrition bias (incomplete outcome data), reporting bias (selective reporting), and other potential sources of bias. Percentages are indicated in parentheses. “Unclear risk” reflects insufficient information to permit a judgment, whereas “high risk” denotes methodological limitations likely to compromise study validity. Notably, heterogeneity in EV characterization and dosing may contribute additional methodological variability not captured by the SYRCLE tool (Supplementary Table S2).

### 3.3 Animal models, ARDS induction, and MSC-EV characteristics

**Figure 2:**
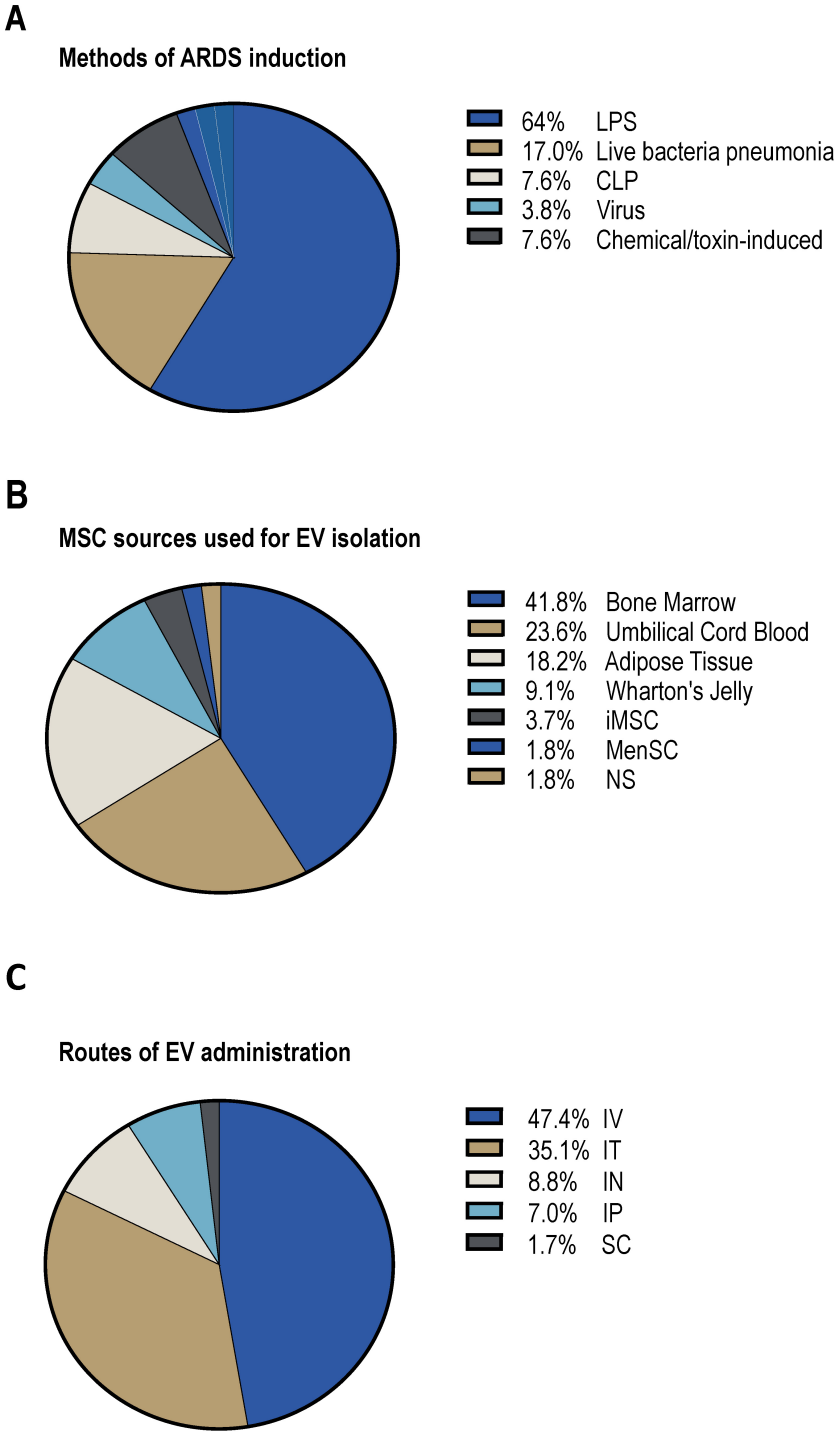
MSC-EVs for the treatment of *in vivo* ARDS. **(A)** Methods of ARDS induction. **(B)** MSC sources used for EV isolation. **(C)** Routes of EV administration. LPS, lipopolysaccharide; CLP, cecal ligation and puncture; iMSC, induced pluripotent stem cell-derived MSCs; MenSC, menstrual blood-derived MSCs; NS, not specified; IV, intravenous; IT, intratracheal; IN, inhalation; IP, intraperitoneal; SC, subcutaneous.

Most included studies employed rodent models, predominantly mice and rats. A minority used large animal models such as pigs, sheep, and Syrian hamsters, particularly in studies involving bacterial or viral ARDS models, to enhance translational relevance. ARDS was induced using diverse methods: intratracheal lipopolysaccharide (LPS, 42%), bacterial pneumonia (commonly *Escherichia coli* or *Pseudomonas aeruginosa*, 17%), cecal ligation and puncture (CLP, 13%), viral infection (e.g., influenza A, SARS-CoV-2, 9%), and less commonly, bleomycin, sulfur mustard, or particulate matter exposure (Figure 2A).

MSC-EVs were derived from bone marrow (BM-MSCs, 40%), umbilical cord (UC-MSCs, 25%), adipose tissue (AD-MSCs, 19%), Wharton's jelly, induced pluripotent stem cell-derived MSCs (iPSC-MSCs), and menstrual blood (Figure 2B). EV characterization adhered variably to MISEV2018 guidelines. The majority reported size distribution via nanoparticle tracking analysis (NTA), morphology via electron microscopy, and surface markers (CD63, CD81, CD9) via immunoblotting or flow cytometry. Most studies administered EVs intravenously (64%), followed by intratracheal (19%), nebulization (11%), and intraperitoneal routes (6%) (Figure 2C).

### 3.4 Therapeutic outcomes and mechanistic insights

Across nearly all included studies, MSC-EV administration conferred robust therapeutic effects in preclinical ARDS models. Key outcomes included reduced histological lung injury, preservation of the alveolar–capillary barrier (reflected by decreased pulmonary edema and protein leakage), and improved gas exchange and survival. These benefits arise from the concerted delivery of a multifaceted cargo of bioactive molecules.

#### 3.4.1 Immunomodulation driven by EV microRNAs and proteins

The immunomodulatory potential of MSC-EVs is well-established. EV treatment consistently reduced neutrophil infiltration and pro-inflammatory cytokines, including tumor necrosis factor-alpha (TNF-α), interleukin (IL)-1β, IL-6, and high-mobility group box 1 (HMGB1), in bronchoalveolar lavage fluid (BALF) and lung tissues (20–24). Mechanistically, MSC-EVs induce macrophage polarization toward an anti-inflammatory M2 phenotype, facilitating resolution of inflammation (25). This effect is mediated by miRNAs, such as miR-146a-5p targeting NF-κB and let-7a modulating TLR4 signaling, as well as by EV-associated proteins, including TNF-stimulated gene 6 (TSG-6) and interleukin-1 receptor antagonist (IL-1RA), which directly antagonize pro-inflammatory pathways.

#### 3.4.2 Tissue repair and barrier restoration via protein and lipid cargo

Beyond immunomodulation, MSC-EVs promote tissue repair and regeneration through protein and lipid cargo. Tight junction preservation (e.g., occludin, claudin-5) and reduced fibrosis are facilitated by growth factors and matrix-modulating proteins, such as angiopoietin-1 (Ang-1), which stabilizes the endothelium, and keratinocyte growth factor (KGF), which drives alveolar epithelial proliferation and barrier restoration (22, 26–28). Lipid components of EVs further enhance endothelial and epithelial barrier function and support cell survival.

#### 3.4.3 Integrated signaling pathways and functional outcomes

Finally, the coordinated modulation of key signaling pathways, including NF-κB and the NLRP3 inflammasome, reflects a synergistic interplay of EV proteins, miRNAs, and surface lipids. This integrated activity improves lung compliance, resolves edema, and limits fibrotic progression. Table 1 summarizes therapeutic and mechanistic outcomes stratified by ARDS induction model.

**Table 1:** Therapeutic effects of MSC-derived EVs in preclinical models of ARDS.

**Injury model (species)**	**Therapeutic outcomes of MSC-EVs**	**Mechanisms**
ARDS [Endotoxin (LPS), acid aspiration, and hemorrhagic shock in rodents]	- Significantly reduced lung inflammation: lower neutrophil counts and decreased levels of TNF-α, IL-1β, IL-6 in BALF and lung tissue (21, 46) - Less alveolar edema and damage: decreased lung wet-to-dry ratio and histological injury score compared to untreated ARDS (23, 46) - Improved pulmonary function: partial restoration of oxygenation and compliance, as well as doubled survival vs. controls (60 vs. 30%) in severe ARDS (21)	- **Macrophage phenotype shift**: EVs promote macrophage polarization to an anti-inflammatory M2 phenotype and reduce neutrophil recruitment (47, 48) - **Inflammation suppression**: EV cargo (e.g., miR-223-3p, miR-27a-3p) inhibits NF-κB signaling and NLRP3 inflammasome activation, leading to reduced cytokine release (21, 36) - **Mitochondrial protection**: EV transfer of mitochondria restores alveolar bioenergetics, improving barrier function (42, 43) - **Endothelial/epithelial barrier support**: EV-treated lungs show upregulated tight junction proteins and less cell apoptosis (25, 28)
Bacterial pneumonia/sepsis (e.g., *E. coli* or *P. aeruginosa pneumonia*, CLP-induced sepsis in rodents; bacterial ARDS in large animals)	- **Improved infection clearance:** EV therapy reduced bacterial load in lung tissue and BALF (e.g., ≥97% reduction in colony-forming units in EV-treated vs. control mice) (20, 49) - **Enhanced survival in sepsis:** in murine sepsis and pneumonia models, MSC-EVs increased survival rates (e.g., 70% survival vs. 30% in controls) (24, 40) - **Reduced inflammatory injury:** EV-treated animals had lower BALF neutrophil count and protein leakage, attenuated cytokine levels, and improved arterial oxygenation in bacterial ARDS (24, 40)	- **Augmented phagocytosis**: EVs boost macrophage and monocyte bacterial phagocytosis. EVs containing enriched miRNAs enhanced the engulfment of bacteria (50) - **Immunomodulation**: EVs modulate leukotriene and immune signaling (e.g., transfer of miR-145 inhibited MRP1, elevating LTB4 levels, aiding in bacterial killing) (50). EV PD-L1 cargo engaged PD-1 on immune cells to dampen excessive inflammation in pneumonia (39)
Viral pneumonia/ARDS (influenza in pigs/mice; COVID-19-like in hamsters)	- **Decreased viral load:** IT MSC-EVs in influenza-infected pigs reduced viral titers in lungs by ~100-fold (51). Treated hamsters showed lower SARS-CoV-2 levels and milder lung pathology than controls (52) - **Mitigation of lung injury:** EV therapy lessened alveolar damage, hemorrhage, and inflammatory cell infiltration in viral ARDS models (51, 52) - **Improved oxygenation and survival:** EV-treated mice infected with severe influenza had higher survival and reduced weight loss/respiratory distress compared to untreated (52)	- **Antiviral cargo**: MSC-EVs carry antiviral miRNAs and proteins that directly impede virus replication (52) - **Cytokine storm modulation**: EV therapy downregulated key cytokines (TNF-α, IL-6, IFN-γ) in virus-injured lungs, preventing hyperinflammation (51, 52)
ARDS-associated early fibrosis	- **Anti-fibrotic effects:** In ARDS-associated early fibrosis, EV-treated mice had significantly lower lung hydroxyproline and fibrosis scores than controls (30) - **Preserved lung function:** EV therapy improved lung compliance, oxygenation (higher PaO_2_/FiO_2_ ratio), and reduced tissue stiffness in fibrotic lung injury models (30) - **Histological improvement:** Treated animals showed better lung architecture with less extracellular matrix accumulation and alveolar thickening (30, 53)	- **Growth factor delivery**: EVs deliver reparative factors like HGF and KGF to injured lungs. EV-derived HGF was essential for anti-fibrotic effects in ARDS-fibrosis, and KGF in MSC-EVs partly mediated improved survival in bacterial pneumonia (20, 30) - **Inhibition of pro-fibrotic pathways**: MSC-EVs interfered with TGF-β/Wnt/β-catenin signaling and epithelial–mesenchymal transition. Treated ARDS mice had lower β-catenin activation and maintained epithelial markers (E-cadherin) *vs*. controls (53)
Toxic inhalation injury (Chemical toxins like sulfur mustard, particulate pollution PM2.5 in rodents)	- **Attenuation of acute lung damage:** hUCMSC-EVs markedly reduced lung injury from sulfur mustard exposure, improving survival and lowering acute inflammation and edema in the lungs. EV-treated animal histology showed less alveolar damage and inflammatory cell infiltration than toxin-only controls (28, 36) - **Protection against oxidative damage:** In PM2.5 smoke exposure, MSC-EVs decreased reactive oxygen species levels and lipid peroxidation in lung tissue, preventing oxidative injury. EV-treated rats had significantly fewer inflammatory cells in BALF and improved lung histology compared to untreated polluted-air exposed rats (54)	- **miR-146a/TLR4-NFκB axis**: EVs delivered miR-146a-5p into mustard gas-injured lungs, suppressing TRAF6, a key adapter in TLR4/NF-κB inflammatory signaling, leading to downregulation of NF-κB and pro-inflammatory cytokines. EVs with enhanced miR-146a showed stronger effects than unmodified EVs (36) - **Antioxidant pathways**: ADMSC-EVs enriched for antioxidant enzymes activated the Nrf2 pathway, increasing lung expression of HO-1, SOD, and catalase, thereby mitigating PM2.5-induced oxidative stress. EV treatment also shifted macrophages toward an M2 phenotype in toxin-exposed lungs (54)

Studies are grouped by injury type, including endotoxin-, acid-, bacterial-, and viral-induced ARDS, early fibrosis, and toxic inhalation injury. For each model, key therapeutic effects of MSC-EVs on inflammation, alveolar–capillary barrier integrity, oxygenation, survival, and tissue repair are summarized. Mechanistic insights encompass immunomodulation (e.g., macrophage polarization, cytokine suppression), miRNA- and protein-mediated signaling, mitochondrial transfer, barrier stabilization, antiviral activity, anti-fibrotic actions, and activation of antioxidant pathways. References indicate representative studies supporting each outcome. ARDS, Acute Respiratory Distress Syndrome; ALI, Acute Lung Injury; BALF, Bronchoalveolar Lavage Fluid; TNF-α, Tumor Necrosis Factor-alpha; IL-1β, Interleukin-1 beta; IL-6, Interleukin-6; HMGB1, High-Mobility Group Box 1; M2, Anti-inflammatory macrophage phenotype; NF-κB, Nuclear Factor kappa-light-chain-enhancer of activated B cells; NLRP3 –, NOD-, LRR, and pyrin domain-containing protein 3; TSG-6, TNF-stimulated Gene 6; IL-1RA, Interleukin-1 Receptor Antagonist; Ang-1, Angiopoietin-1; KGF, Keratinocyte Growth Factor; CLP, Cecal Ligation and Puncture; IT, Intratracheal; HGF, Hepatocyte Growth Factor; TGF-β, Transforming Growth Factor-beta; HO-1, Heme Oxygenase-1; SOD, Superoxide Dismutase; ADMSC, Adipose-Derived Mesenchymal Stromal Cell; hUCMSC, Human Umbilical Cord Mesenchymal Stromal Cell; PM2.5, Particulate Matter ≤ 2.5 μm.

### 3.5 Comparative outcomes across ARDS models

Despite the heterogeneity of ARDS models, the anti-inflammatory effects of MSC-EVs were consistently observed. This was accompanied by enhanced expression of IL-10, Vascular Endothelial Growth Factor (VEGF), and antioxidant enzymes [e.g., Superoxide Dismutase (SOD), catalase], with downstream improvements in oxygenation (PaO_2_/FiO_2_), lung histology, and survival (25, 29, 30).

### 3.6 Survival benefits and safety profile

Several studies reported improved survival following MSC-EV administration. Enhanced survival was observed with both naïve and IFN-γ-primed EVs in *E. coli*–induced pneumonia (31). Importantly, safety evaluations revealed no adverse events or organ toxicity, even with high-dose or repeated administration. In a Good Laboratory Practice (GLP)-compliant toxicology study, no evidence of systemic or pulmonary toxicity was found in rats treated with high-dose inhaled MSC-EVs for 28 days (32). Biodistribution studies indicated rapid clearance and no long-term EV engraftment, supporting a transient, paracrine mode of action (33).

### 3.7 Influence of MSC-EV source and preconditioning

Therapeutic efficacy varied with the cellular source and conditioning of MSCs. AD-MSC-derived EVs demonstrated superior anti-inflammatory effects in sepsis-associated ARDS models compared to BM- or UC-MSC-EVs (34). Donor age also influenced efficacy, with EVs from younger donors outperforming those from aged sources (35). Preconditioning MSCs with LPS, IFN-γ, or thrombin enhanced EV potency, with IFN-γ-primed EVs exerting particularly strong immunomodulatory effects in endotoxin-induced ARDS (23). Bioengineered EVs enriched with therapeutic microRNAs (e.g., miR-181a-5p, miR-146a-5p) or proteins (e.g., PD-L1) further improved bacterial clearance and mitigated cytokine storms in severe infection models (31, 32, 36, 37).

MSC-EV efficacy was also affected by injury severity and route of administration. Single-dose therapy was often sufficient in mild-to-moderate ARDS, whereas severe models, such as CLP-induced sepsis, typically required higher or repeated dosing. In a large-animal model of bacterial pneumonia-induced ARDS, a single intravenous dose failed to improve physiological outcomes, emphasizing the need for species- and model-specific optimization of delivery strategies (38).

### 3.8 Delivery route considerations

The route of administration strongly affected EV distribution and therapeutic efficacy. Pulmonary delivery, through intratracheal instillation or nebulization, increased EV availability in the lungs while reducing systemic clearance. This localized delivery raised EV concentrations at the site of injury, improving control of lung inflammation and promoting repair of the alveolar–capillary barrier. Studies consistently found that pulmonary delivery provided equal or superior outcomes—such as reduced edema and better oxygenation, compared with intravenous injection, often requiring lower doses and posing less risk of systemic immune effects (22, 32, 39).

## 4 Discussion

This systematic review demonstrates that MSC-EVs consistently confer therapeutic benefits in preclinical ARDS models. Administration of MSC-EVs attenuated inflammation, preserved alveolar–capillary barrier integrity, improved oxygenation and lung histopathology, and, in some studies, enhanced survival. These effects were observed across bacterial, viral, chemical, and sepsis-induced models, highlighting the broad applicability of MSC-EV therapies.

Mechanistically, MSC-EVs deliver a complex repertoire of bioactive molecules—including microRNAs, proteins, lipids, mRNAs, and mitochondria—that collectively modulate immune responses, promote tissue repair, and restore pulmonary homeostasis. Their immunomodulatory actions involve suppression of proinflammatory signaling and polarization of alveolar macrophages toward an M2 phenotype, as reflected by reduced TNF-α and iNOS expression and increased CD206 expression and phagocytic activity (25). Several EV-associated microRNAs, including miR-181a-5p, miR-27a-3p, miR-223-3p, and miR-146a-5p, regulate inflammatory pathways by targeting PTEN/SOCS1 and NF-κB/TRAF6 signaling cascade (21, 36, 37, 40, 41).

Beyond microRNAs, EV proteins such as TNF-stimulated gene 6, IL-1 receptor antagonist, angiopoietin-1, and keratinocyte growth factor contribute to endothelial stabilization, epithelial regeneration, angiogenesis, and antifibrotic remodeling (5, 30, 42–45). Lipid constituents of EV membranes enhance barrier integrity, cell survival, and repair capacity, while mitochondrial transfer restores cellular bioenergetics. Collectively, these interconnected mechanisms target key pathophysiological processes in ARDS—including immune dysregulation, alveolar–endothelial injury, impaired pathogen clearance, and fibrotic remodeling—underscoring the multifactorial therapeutic potential of MSC-EVs (5).

### 4.1 Challenges and translational limitations

Despite these promising findings, several limitations constrain interpretation and translation. Many studies lacked rigorous controls (e.g., heat-inactivated EVs, EV-depleted media, or non-MSC EVs), complicating attribution of observed effects specifically to EV cargo. Regenerative outcomes, including epithelial and endothelial repair and angiogenesis, were infrequently assessed. Model-specific variability was evident: large-animal ovine sepsis studies often failed to replicate rodent findings, likely due to differences in dosing, pulmonary distribution, or timing of administration (24, 38, 40). Donor age also affected efficacy, with EVs from aged MSCs exhibiting reduced potency and altered cargo profiles (e.g., elevated pro-inflammatory miRNAs, reduced miR-223) (35), whereas iPSC-MSCs produced consistent and robust EVs in sepsis and endotoxemia models (26, 40).

Safety data remain limited, with formal toxicity assessments, dose-escalation protocols, and long-term follow-up largely absent. While repeated dosing improved outcomes in some studies (46), systematic evaluation of dose-dependent toxicity, immune activation, or off-target organ effects is lacking. Progress in EV manufacturing is encouraging. Good manufacturing practice (GMP)-compliant protocols and stability data now support long-term storage of clinical-grade EVs (11, 32). Aerosolized delivery via nebulization has shown comparable or superior efficacy to intravenous administration in pneumonia models, offering a non-invasive approach for targeted pulmonary therapy, particularly in ventilated patients (32).

Translational relevance is limited by the predominant use of young, otherwise healthy animals with acute injury, which does not capture the complexity of human ARDS, where advanced age, comorbidities, and chronic lung damage are common (1). Moreover, substantial variability exists in EV characterization, dosing, and reporting. Administered doses ranged from 1 × 106 to 3 × 10^9^ particles per mouse (median ~ 4.3 × 10), and quantification methods varied between particle counts and protein content, hindering cross-study comparisons. Establishing consensus on dosing metrics, potency assays, and reporting standards is essential to improve reproducibility and accelerate clinical translation.

Bioengineering approaches offer promising strategies to enhance MSC-EV efficacy. Preconditioning MSCs (e.g., with hypoxia or inflammatory stimuli), genetic modification, and selective cargo enrichment have all demonstrated improved therapeutic outcomes. For instance, let-7a-5p–enriched EVs mitigated fibrosis, while EVs from HSF1-overexpressing MSCs increased survival in hemorrhagic shock models (28, 30). Combining MSC-EVs with standard ARDS therapies, such as corticosteroids, antibiotics, or antifibrotic agents, warrants exploration. Notably, early-phase ARDS models show the greatest responsiveness to EV therapy, highlighting a potential therapeutic window for early intervention.

### 4.2 Study limitations

A key limitation of this review is the absence of prospective protocol registration in a public database such as PROSPERO. This decision reflected the exploratory and rapidly evolving nature of the preclinical literature on MSC-derived extracellular vesicles (MSC-EVs) in ARDS. The main objective was to systematically map emerging evidence, delineate mechanistic insights, and identify knowledge gaps, rather than to address a narrowly defined clinical question—criteria more suited to PROSPERO registration. Furthermore, PROSPERO primarily supports clinical systematic reviews and is not fully optimized for preclinical or animal research. The dynamic evolution of this field required iterative adjustments to the review protocol to include novel experimental models, EV characterization techniques, and mechanistic endpoints. While these adaptations ensured comprehensive coverage, they precluded a fixed, pre-registered design. Nevertheless, all stages of the review were conducted according to a rigorous, internally documented protocol, with consistent methodology applied to minimize bias. This limitation has been explicitly acknowledged to enhance transparency and guide interpretation of the findings. Future systematic reviews should consider prospective protocol registration (e.g., in PROSPERO or OSF), adhere to PRISMA recommendations, and predefine primary and secondary outcomes as well as analytic strategies. Such practices will further strengthen transparency, reduce selective reporting, and enhance comparability across studies

Although we explored opportunities for quantitative synthesis, a meta-analysis was not performed due to pronounced heterogeneity in (i) experimental models and species (mouse, rat, pig, sheep, hamster), (ii) injury mechanisms (endotoxin, bacterial or viral pneumonia, toxic inhalation, fibrosis-associated injury), (iii) EV sources, engineering strategies, dosing, and administration routes (intravenous, intratracheal, or nebulized), and (iv) outcome timing (ranging from 6 to 72 h and beyond). This heterogeneity, together with incomplete reporting of summary statistics (means ± SDs or events/denominators) and misaligned timepoints, precluded a defensible pooled estimate across three or more comparable studies for the same endpoint and time window. We acknowledge that the absence of a quantitative synthesis represents a limitation. To enable future meta-analyses, we recommend harmonized reporting for outcomes most amenable to pooling—such as survival, oxygenation indices, bronchoalveolar lavage protein, and key cytokines (e.g., IL-6)—with standardized timepoints (24 ± 3 h and 48 ± 6 h), complete summary statistics [mean ± SD or median (interquartile range) with transformation], and clear specification of EV dose and administration route.

## 5 Concluding remarks and future directions

Preclinical evidence firmly establishes MSC-EVs as a next-generation therapeutic for ARDS. Their efficacy arises from pleiotropic immunomodulatory, reparative, and antifibrotic actions, and their acellular nature offers an inherently favorable safety profile, making them a compelling alternative to whole-cell therapies. Translating these findings into clinical practice, however, requires a coordinated strategy to overcome key translational challenges. Foremost, the field must address inconsistencies that limit reproducibility and cross-study comparability. Standardized protocols for EV characterization, quantification, and reporting are essential. Mechanistic studies should progress beyond descriptive observations to delineate causative pathways using advanced molecular and functional assays, incorporating regenerative endpoints such as epithelial proliferation, endothelial repair, and angiogenesis.

To accelerate clinical translation, we propose a three-pronged roadmap: (1) Scalable manufacturing: Develop closed, bioreactor-based, GMP-compliant systems to ensure reproducible, large-scale production of clinical-grade EVs with defined critical quality attributes. (2) Functional enhancement: Employ bioengineering approaches, including parental cell preconditioning (e.g., hypoxia, 3D culture) or direct EV modification, to enhance tissue targeting, enrich therapeutic cargo (e.g., anti-inflammatory microRNAs, angiogenic proteins), and generate precision-engineered vesicles tailored to ARDS pathophysiology, and (3) Clinical integration: Position MSC-EVs within multimodal ARDS management by identifying synergistic interactions with standard supportive therapies (e.g., lung-protective ventilation) and defining patient endotypes most likely to benefit, laying the foundation for personalized EV-based interventions.

By combining rigorous mechanistic insight, scalable production, and strategic clinical deployment, MSC-EV therapy has the potential to transition from a promising preclinical concept to a transformative, patient-centered intervention, ultimately improving outcomes in ARDS and related acute lung injuries.

## Data Availability

The raw data supporting the conclusions of this article will be made available by the authors, without undue reservation.
