# Supplementary material for: Extracellular vesicles from mesenchymal stromal cells as a promising therapy for ARDS: a systematic review of preclinical studies
Source: Front Med (Lausanne). 2025 Oct 29;12:1665948. doi: 10.3389/fmed.2025.1665948 (PMC12605180; doi:10.3389/fmed.2025.1665948)
Supplement: Supplementary file 1 [file Table_1.docx]

# **Table S1. Summary of Included Studies — Extracted Data and Access Links**

| **DOI** | **Animal** | **Model** | **Route of administration** | **Route of administration** | **MSC source** | **EV dose** |
| --- | --- | --- | --- | --- | --- | --- |
| 10.1016/j.intimp.2023.110693 | mouse | Hemorrhagic shock | IP | IN | BM | 10x10^9^/kg |
| 10.1164/rccm.201701-0170OC | mouse | LPS | IN | IN | BM |  |
| 10.3390/ijms23073417 | mouse | LPS | IN | IN | UCB |  |
| 10.1155/2023/8987049 | rat | CLP sepsis model |  | IP | iMSC | 2 mg/kg |
| 10.3390/cells12232729 | mouse | LPS | IN | IP | UCB |  |
| 10.1007/s12015-024-10784-6 | mouse | LPS | IP | IP | UCB | 3x10^8^ |
| 10.1186/s13287-024-03708-1 | rat | LPS | IT | IT | AD | 0.6-15x10^7^ |
| 10.1002/ctm2.287 | mouse | MDR-PA pneumonia | IT | IT | AD | 10 ug |
| 10.1016/j.tox.2020.152627 | rat | PM2.5 | IT | IT | AD | 3x10^10^ |
| 10.3389/fmed.2023.1162615 | rat | *E. coli* | IT | IT | BM |  |
| 10.2147/IJN.S485012 | mouse | LPS | IT | IT | BM | 1x10^10^ |
| 10.3390/pharmaceutics16101316 | rat | LPS | IT | IT | BM | 1x10^8^ |
| 10.1186/s13287-021-02143-w | mouse | LPS | IT | IT | BM | 0.1x10^10^ |
| 10.1021/acsnano.1c03231 | mouse | LPS | IP | IT | BM | 6x10^7^/20 gbw |
| 10.1002/stem.2619 | mouse | PA LPS | IT | IT | BM |  |
| 10.1186/s13287-018-0774-8 | Pig | SwIV | IN | IT | BM | 80 ug/kg/bw |
| 10.1155/2022/7837837 | mouse | LPS | IP | IT | BM,UCB, and AD | 100 μg |
| 10.1016/j.ymthe.2023.01.025 | mouse | LPS | IT | IT | MenSC |  |
| 10.1186/s13062-024-00586-8 | mouse | LPS | IT | IT | Not specified | 50 μg |
| 10.1111/cpr.13531 | mouse | LPS | IT | IT | UCB |  |
| 10.1016/j.biocel.2019.05.010 | rat | Bleomycin | IT | IT | WJ |  |
| 10.1186/s13287-020-01617-7 | rat | Bleomycin | IT | IT | WJ | 1x10^6^ |
| 10.1186/s12931-024-02908-w | mouse | *E. coli* | IT | IT | WJ | 1x10^9^ |
| 10.1097/SHK.0000000000002381 | mouse | LPS | IP and IT | IT IP and IV | iMSC | 0.6 mg/kg |
| 10.1164/rccm.201410-1765OC | mouse | *E. coli* | IT | IT or IV | BM |  |
| 10.1021/acsbiomaterials.3c01173 | mouse | LPS | IT | IT or IV | BM |  |
| 10.1016/j.intimp.2024.111519 | mouse | CLP sepsis model |  | IV | AD | 200 μg |
| 10.1021/acsbiomaterials.3c00614 | mouse | CLP sepsis model |  | IV | AD | 100 μg |
| 10.18632/aging.102314 | mouse | LPS | IT | IV | AD | 100 μg |
| 10.1097/CCM.0000000000004315 | mouse | LPS | IT | IV | AD | 50 μg |
| 10.1002/jev2.12495 | hamster | SARS-CoV-2 | IN | IV | AD | 3x10^9^ and 1x10^10^ |
| 10.4049/jimmunol.1801534 | mouse | *E. coli* | IT | IV | BM | 1x10^10^ |
| 10.1136/thoraxjnl-2021-218194 | mouse | LPS | IT | IV | BM |  |
| 10.1183/13993003.02978-2020 | mouse | LPS | IT | IV | BM |  |
| 10.2147/DDDT.S344309 | mouse | LPS | IT | IV | BM |  |
| 10.3390/ijms23095196 | mouse | LPS | IP | IV | BM |  |
| 10.1371/journal.pone.0259732 | rat | LPS | IT | IV | BM |  |
| 10.1155/2019/8262849 | mouse | LPS | IT or IP | IV | BM |  |
| 10.4252/wjsc.v16.i8.811 | mouse | LPS | IN and IP | IV | BM | 100 μg |
| 10.3389/fimmu.2023.1136964 | sheep | *P. aeruginosa* | IT | IV | BM |  |
| 10.1186/s13287-021-02329-2 | mouse | PA103 | IT | IV | BM |  |
| 10.1097/ALN.0000000000002655 | rat | *E. coli* | IT | IV | UCB |  |
| 10.1007/s12013-023-01208-2 | mouse | LPS | IT | IV | UCB |  |
| 10.1016/j.jcyt.2024.04.074 | mouse | LPS | IT | IV | UCB | 60 μg |
| 10.1186/s12951-023-02038-3 | mouse | LPS | IT | IV | UCB | 40 μg |
| 10.1186/s13062-022-00351-9 | mouse | LPS | IT | IV | UCB | 0.9x10^9^ |
| 10.1002/jcb.30519 | mouse | LPS | IT | IV | UCB |  |
| 10.1016/j.jcyt.2021.05.009 | mouse | CLP sepsis model |  | IV | WJ |  |
| 10.1186/s12929-024-01019-4 | rat | hyperoxia |  | IV | WJ | 2x10^10^ |
| 10.1186/s13287-023-03385-6 | mouse and rat | LPS and *E. coli* | IT | IV or IN | BM and UCB | 5x10^8^-5x10^9^ |
| 10.1186/s13287-023-03375-8 | mouse | sulfur mustard | Subcutaneous (back) | Subcutaneous | UCB | 3x10^8^ |

IP, intraperitoneal; IV, intravenous; IN, inhalation; IT, intratracheal; AD, adipose-derived; BM, bone marrow; UCB, umbilical cord blood; iMSC, iPSC-derived MSC; MenSC, menstrual blood MSC; WJ, Wharton’s Jelly; LPS, lipopolysaccharide; CLP, cecal ligation punction; PM2.5, particular matter 2.5; MDR-PA, multidrug-resistant *Pseudomonas aeruginosa*; PA LPS, *P. aeruginosa*  lipopolysaccharide SwIV, swine influenza virus; *E. coli*, *Escherichia coli*; SARS-CoV-2, Severe acute respiratory syndrome coronavirus 2; PA-103; *P. aeruginosa*  PA-103.
