# Supplementary material for: Extracellular vesicles from mesenchymal stromal cells as a promising therapy for ARDS: a systematic review of preclinical studies
Source: Front Med (Lausanne). 2025 Oct 29;12:1665948. doi: 10.3389/fmed.2025.1665948 (PMC12605180; doi:10.3389/fmed.2025.1665948)
Supplement: Supplementary file 2 [file Table_2.docx]

**Table S2.** **Risk-of-bias assessment of included preclinical studies (n = 51) using the SYRCLE tool.**

| **Domain** | **Low Risk** | **Unclear Risk** | **High Risk** | **Notes** |
| --- | --- | --- | --- | --- |
| Sequence generation (randomization) | 8 (16%) | 43 (84%) | 0 | Only a minority reported explicit random allocation |
| Baseline characteristics | 50 (98%) | 1 (2%) | 0 | Most studies reported comparable baseline conditions |
| Allocation concealment | 0 | 51 (100%) | 0 | No study reported allocation concealment |
| Random housing (performance bias) | 5 (10%) | 30 (59%) | 16 (31%) | Random housing rarely described; some high risk |
| Blinding of caregivers/investigators (performance bias) | 0 | 20 (39%) | 31 (61%) | Blinding not reported in most studies |
| Blinding of outcome assessment (detection bias) | 0 | 45 (88%) | 6 (12%) | Mostly unclear; few high risk |
| Incomplete outcome data (attrition bias) | 50 (98%) | 1 (2%) | 0 | Nearly all studies reported complete data |
| Selective reporting (reporting bias) | 0 | 46 (90%) | 5 (10%) | Protocols unavailable; selective reporting cannot be excluded |
| Other sources of bias | 51 (100%) | 0 | 0 | No major concerns, though EV characterization/dose variability may be an issue |

The table summarizes the proportion of studies rated as low, unclear, or high risk across nine domains: sequence generation, baseline characteristics, allocation concealment, performance bias (random housing and blinding of caregivers/investigators), detection bias (blinding of outcome assessment), attrition bias (incomplete outcome data), reporting bias (selective reporting), and other sources of bias. Percentages are shown in parentheses. “Unclear risk” indicates insufficient information to judge the domain, while “high risk” indicates potential bias that may affect study validity. Variability in extracellular vesicle characterization and dose reporting may represent additional methodological heterogeneity not captured by the tool.
